# Protective Effect of Boswellic Acids against Doxorubicin-Induced Hepatotoxicity: Impact on Nrf2/HO-1 Defense Pathway

**DOI:** 10.1155/2018/8296451

**Published:** 2018-02-06

**Authors:** Bassant M. Barakat, Hebatalla I. Ahmed, Hoda I. Bahr, Alaaeldeen M. Elbahaie

**Affiliations:** ^1^Department of Pharmacology and Toxicology, Faculty of Pharmacy (Girls), Al-Azhar University, Cairo, Egypt; ^2^Department of Biochemistry, Faculty of Veterinary Medicine, Suez Canal University, Ismailia, Egypt; ^3^Department of Clinical Oncology and Nuclear Medicine, Faculty of Medicine, Suez Canal University, Ismailia, Egypt

## Abstract

The current study aimed to investigate the potential protective role of boswellic acids (BAs) against doxorubicin- (DOX-) induced hepatotoxicity. Also, the possible mechanisms underlying this protection; antioxidant, as well as the modulatory effect on the Nrf2 transcription factor/hem oxygenase-1 (Nrf2/HO-1) pathway in liver tissues, was investigated. Animals were allocated to five groups: group 1: the saline control, group 2: the DOX group, animals received DOX (6 mg/kg, i.p.) weekly for a period of three weeks, and groups 3–5: animals received DOX (6 mg/kg, i.p.) weekly and received protective doses of BAs (125, 250, and 500 mg/kg/day). Treatment with BAs significantly improved the altered liver enzyme activities and oxidative stress markers. This was coupled with significant improvement in liver histopathological features. BAs increased the Nrf2 and HO-1 expression, which provided protection against DOX-induced oxidative insult. The present results demonstrated that BAs appear to scavenge ROS and inhibit lipid peroxidation and DNA damage of DOX-induced hepatotoxicity. The antioxidant efficacy of BAs might arise from its modulation of the Nrf2/HO-1 pathway and thereby protected liver from DOX-induced oxidative injury.

## 1. Introduction

Organ dysfunction is common within cancer patients, and hepatic dysfunction has a great impact on the patient outcome leading to a health-care burden [1]. Doxorubicin (DOX) is a widely used chemotherapeutic agent, possessing a broad spectrum of antineoplastic effects against various tumor types and hematological malignancies [2]. Although DOX is a successful cancer chemotherapeutic, side effects limit the clinical utility of DOX-based therapy, including dose-dependent chronic cardiotoxicity, myelosuppression [3], and hepatotoxicity [4]. While the full mechanism of DOX-related cytotoxicities is not fully understood, it has been demonstrated that oxidative stress plays an essential role in DOX-induced toxicity [5]. Reactive oxygen species (ROS) can damage liver membranes causing the release of liver enzymes. Thus, controlling the oxidative injury by different agents is extensively appreciated.

Recently, the protective properties of pentacyclic triterpenoids have gained increasing attention. For instance, oleanolic and ursolic acids are triterpenoids extensively distributed in food and remedial plants [6] and exhibiting protective properties against liver injury in experimental models [7]. Additionally, it was documented that ursolic acid promotes neuroprotection after cerebral ischemia in mice by triggering the NF-E2-related factor 2 (Nrf2) pathway [8].

*Boswellia serrata* resin extracts show an antioxidant activity in a diversity of experimental diseases including ulcerative colitis [9], myocardial I/R injury [10], and pulmonic fibrosis [11]. Acetyl 11-keto-b-boswellic acid (AKBA) is a pentacyclic triterpenoid compound. It is known as the most significant component of *Boswellia serrata* resin [12]. AKBA has a structural similarity to ursolic acid, and interestingly, one study affirmed that AKBA has greater antioxidant activity in mice than ursolic acid [13]. Consequently, it may promote some of its protective effects via activating the Nrf2 pathway, a hypothesis which was proved recently by Zhang et al. [14].

It was shown that Nrf2 is involved in regulating the expression of genes encoding antioxidant proteins and detoxifying enzymes of phase 2 through a promoter sequence termed the antioxidant response element. The importance of Nrf2 and its downstream proteins such as NAD(P)H, glutathione S-transferases, and heme oxygenase-1 (HO-1) has been evidenced in guarding against chemically induced oxidative stress causing cellular insult in several organs [15–17]. Among these genes, many studies have concentrated on the regulation and action of HO-1 as it has been proven to have the highest antioxidant response elements on its promoter. HO-1 catalyzes the first and rate-limiting step in heme breakdown and generation of the antioxidants biliverdin and bilirubin [18, 19].

Taking altogether, the profitable features of triterpenoids and the probable role of the Nrf2/HO-1 pathway, our hypothesis is that BAs may offer a protective effect against hepatotoxicity of DOX. We tested if this putative protective effect involves the stimulation of the Nrf2/HO-1 pathway.

## 2. Materials and Methods

### 2.1. Chemicals and Drugs

A standardized *B. serrata* extract containing 65% BAs in the form of a tablet preparation was purchased from Advance Physician Formulas Inc. (California, USA). Tablets were grid and suspended in distilled water. Adricin vial containing 2 mg/ml of DOX was obtained from EIMC United Pharmaceuticals (Cairo, Egypt), and dilution was done with saline solution.

### 2.2. Animals

Animal experiments were licensed and performed according to the rules of the research ethics committee at the Faculty of Pharmacy, Al-Azhar University, and complied with the National Institutes of Health Guide for the Care and Use of Laboratory Animals. We used male Swiss albino mice weighing 21–25 g. Mice were fed a regular chow diet with water ad libitum and maintained in a clean room with normal light and dark cycles. Animals were purchased from the Modern Veterinary Office in Cairo.

### 2.3. Design of the Experiment

Mice were equally and randomly assigned into five groups, eight animals per group: group 1: animals were injected with saline weekly [16 ml/kg, i.p.] and received distilled water [12 ml/kg/day] orally; group 2 (DOX group): DOX was given to animals every week (6 mg/kg, i.p.) [20] in addition to distilled water [12 ml/kg/day p.o.]; and groups 3, 4, and 5: animals received DOX (6 mg/kg, i.p.) every week and were treated with three doses of BAs [125, 250, and 500 mg/kg/day, p.o.]. All treatment regimens were continued for 3 weeks.

The intraperitoneal route was used for injecting DOX and saline weekly in a volume of 16 ml/kg whereas the oral gavage needle was used to administer BAs and distilled water daily in a total volume of 12 ml/kg. Generally, DOX or saline was given at days 1, 8, and 15 of the experiment. However, BAs started on day 1 and extended to day 21.

### 2.4. Blood and Sample Collection

At the last day of the experiment, mice were sacrificed by cervical dislocation after being anesthetized with ketamine (80 mg/kg, i.p.). Samples of blood were collected in dry tubes and centrifuged at 1200 ×g. Serum samples were then collected in clean Eppendorf tubes and kept at −20°C to be utilized later in biochemical assays.

For the evaluation of liver function, serum alanine aminotransferase (ALT) and aspartate aminotransferase (AST) activities were determined by enzymatic colorimetric kits from Biodiagnostic (Egypt). Readings for the reaction colors were obtained using an ultraviolet-visible spectrophotometer (UV-1601-PC; Shimadzu, Japan).

Then, each mouse abdomen was opened, and liver was separated and washed in cold phosphate-buffered saline (pH = 7.4). One liver portion was utilized for DNA extraction used in DNA laddering assay, and another one was stored at −80°C for the preparation of tissue homogenate (prepared as 10% *w*/*v*).

### 2.5. Determination of Oxidative Stress Markers

Frozen liver samples were homogenized in phosphate-buffered saline (pH 7.4) by the aid of a Teflon homogenizer. Tissue homogenates were then put in a cooling centrifuge at 2000 ×g for 10 min. The supernatants were used for malondialdehyde (MDA) determination which has been identified as the product of lipid peroxidation that reacts with thiobarbituric acid (TBA) in acidic medium at 95°C to give pink product measured by a spectrophotometer at wave length equals 534 nm using tetramethoxypropane as a standard [21, 22].

### 2.6. Western Blot Analysis for Nrf2, HO-1, and Cleaved Caspase-3

The frozen tissues were homogenized and lysed using ice-cold lysis buffer (10% glycerol, 2% SDS in 62 mM Tris-HCl, pH 6.8) containing a cocktail of protease inhibitors (Sigma-Aldrich, St. Louis, MO). The protein content in the collected protein lysates was quantified using the Bradford method [23]. Equal amounts of total protein were resolved under denaturing conditions by sodium dodecyl sulfate-polyacrylamide gel electrophoresis (SDS-PAGE) and then transferred into nitrocellulose membranes. Blocking was done with 6% non-fat dry milk in TBS-Tween buffer for 3 h at 4°C; then the nitrocellulose membranes were incubated at 4°C overnight with the primary antibodies against the target proteins (Nrf2, HO-1, and cleaved caspase-3). For Nrf2, we used Human/Mouse/Rat Nrf2 Antibody Monoclonal Mouse IgG2B Clone number 383727 from R and D Systems Inc. (Minneapolis, USA), for HO-1, we used (A-3): sc-136960 antibody from Santa Cruz Biotechnology Inc. (Dallas, Texas, USA), and for cleaved caspase-3, we used cleaved caspase-3 (Asp175) antibody from Cell Signaling Technology (Danvers, MA, USA). Again, the membranes were incubated with *β*-actin monoclonal antibody for 1 h on a roller shaker at 4°C. Membranes were then washed 5 times (5 min each) in TBS-Tween buffer in order to get rid of the excess primary antibodies after which they were incubated with an appropriate horseradish peroxidase-conjugated secondary antibodies for another 1 h at 37°C. The bands were scanned using ChemiDoc scanner, and then the densitometric intensity of each band was quantified.

### 2.7. DNA Fragmentation Analysis for Detecting Apoptosis

The liver gnomic DNA was extracted by a Bio Basic EZ-10 spin column genomic DNA kit (Markham, Canada). The diluted samples of extracted genomic DNA (90 ng/ml) were resolved by 0.8% (*w*/*v*) agarose gel electrophoresis for 2 h (90 V and 110 mA) and visualized by ethidium bromide staining using UV illumination. The 100 bp DNA ladder, a ready to use molecular weight marker, was purchased from Solis Biodyne (Tartu, Estonia). The gel was imaged using a gel documentation system; then analysis was done using Gel Docu advanced version 2 software.

### 2.8. Histopathological Examination

For the investigation of histopathological abnormalities, specimens were taken from the biggest lobe of the liver, fixed with 10% formaldehyde, and embedded in paraffin. From these paraffin blocks, tissue sections were cut and stained with hematoxylin and eosin (H and E). Histological examination for hepatic tissues was done by an expert pathologist who was blinded to the experimental groups. Abnormal histopathological findings were evaluated using a semiquantitative method according to standards proposed by the Knodell histology activity index scoring system [24]. In brief, it grades necroinflammation and fibrosis on a scale of 0 to 22, including 0 to10 for the periportal and/or bridging necrosis, 0 to 4 for intralobular degeneration and focal necrosis, 0 to 4 for portal inflammation, and 0 to 4 for fibrosis.

### 2.9. Statistical Analysis of the Results

Data were expressed as the mean ± SD and analyzed using the Statistical Package of Social Sciences (Chicago, IL, USA). Differences among means were tested for significant differences employing one-way analysis of variance (ANOVA) test followed by Bonferroni's multiple comparison test in order to determine differences between each pair of study groups. Statistical significance was considered at *P* < 0.05.

## 3. Results

Results indicated that injecting DOX in a dose of 6 mg/kg weekly increased serum liver enzyme level; ALT and AST activities increased to 158.83 ± 11.49 and 191.33 ± 18.48 versus 59.17 ± 5.87 and 84.17 ± 7.11 in group 1 (saline control), respectively. Combining BAs with DOX (groups 3, 4, and 5) reduced the serum liver enzyme activities in comparison to group 2 (DOX control). The effect of BAs (500 mg/kg) was significantly different from the lowest dose (125 mg/kg) (Table 1).

**Figure 1:**
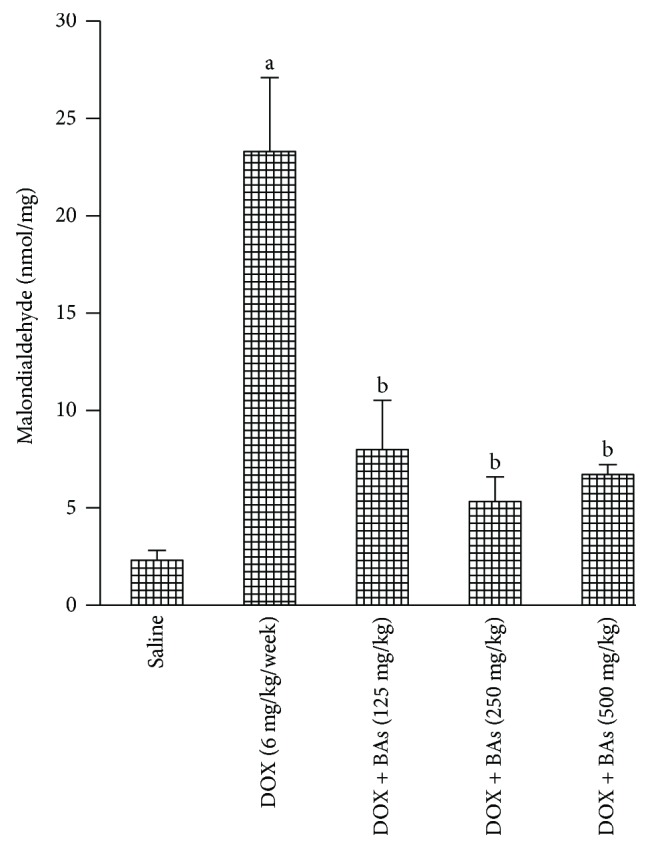
Malondialdehyde level in liver tissue in experimental groups. Mice were injected with doxorubicin (DOX, 18 mg/kg i.p.) in combination with boswellic acids (BAs, 125, 250, or 500 mg/kg). Data are the mean ± SD, and analysis was done by one-way ANOVA followed by Tukey's post hoc test. ^a^*P* value < 0.05 compared to the saline group; ^b^*P* value < 0.05 compared to the DOX group.

Furthermore, hepatic MDA levels increased 10-fold in group 2 (DOX control) as compared with group 1 (saline control). Therapeutic doses of BAs (groups 3, 4, and 5) decreased the MDA level to one-third of the value recorded in group 1 (DOX control) (Figure 1).

**Figure 2:**
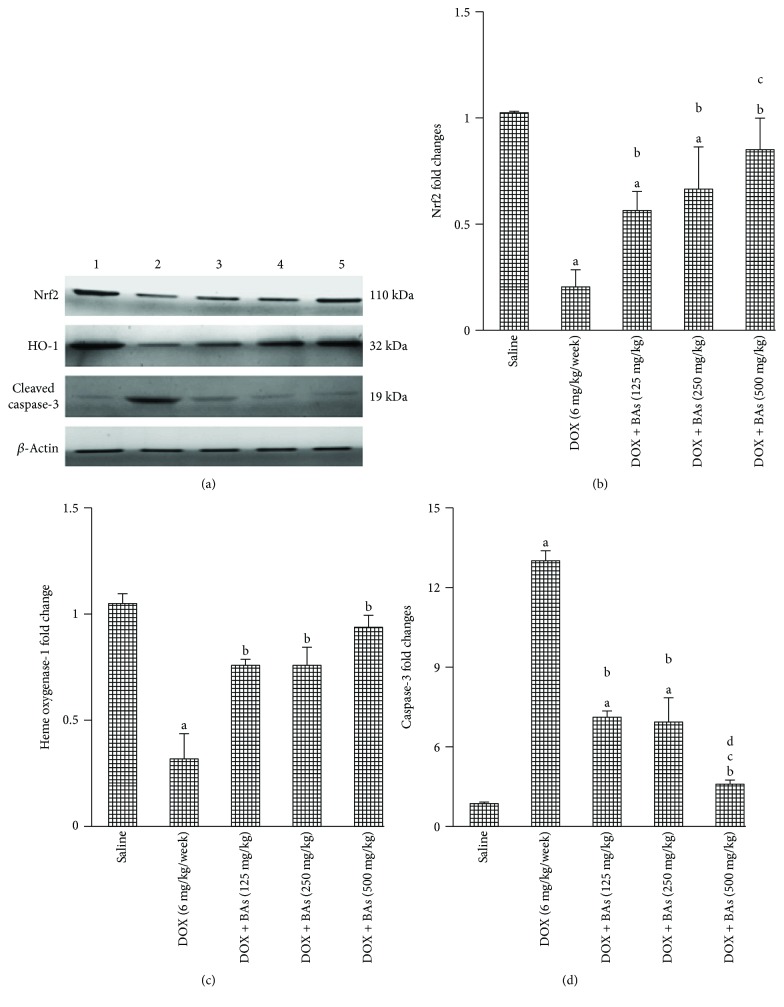
Western blot analysis for Nrf2, heme oxygenase-1, and cleaved caspase-3. (a) Western blots for the measured factors in comparison to *β*-actin, lane 1 (saline), lane 2 (DOX control), and lanes 3, 4, and 5 (DOX in combination with BAs (125, 250, or 500 mg/kg). Column charts for fold changes in Nrf2 (b), heme oxygenase-1 (c), and cleaved caspase-3 (d). DOX: doxorubicin; BAs: boswellic acids; HO-1: heme oxygenase-1. Data are the mean ± SD, and analysis was done by one-way ANOVA followed by Tukey's post hoc test. ^a^*P* value < 0.05 compared to the saline group, ^b^*P* value < 0.05 compared to the DOX group, ^c^*P* value < 0.05 compared to the doxorubicin + BAs (125 mg/kg) group, and ^d^*P* value < 0.05 compared to the doxorubicin + BAs (250 mg/kg) group.

Western blot analysis indicated downregulation in genes encoding for Nrf2 and HO-1 {0.2 and 0.3 of the value reported with group 1 (saline control)} (Figures 2(a)–2(c)) and upregulation in gene encoding for cleaved caspase-3 in group 2 (DOX control—12.45-fold increase) versus group 1 (saline control) (Figures 2(a) and 2(d)). Coadministration of BAs with DOX increased the expression of Nrf2 gene in comparison to the DOX control group. The expression of Nrf2 gene in group 5 (DOX + BAs/500 mg/kg) was significantly greater than that in group 3 (DOX + BAs/125 mg/kg). Similarly, these combinations upregulated HO-1 compared to group 2 (DOX control) (Figures 2(b) and 2(c)). Further, the addition of BAs in all used doses (groups 3, 4, and 5) to DOX regimen differentially downregulated cleaved caspase-3 expression if compared to the DOX control group (group 2). Further, BAs/500 mg/kg/day (group 5) downregulated cleaved caspase-3 expression to a greater extent compared to either of the lower doses (groups 3 and 4) when added to DOX regimen (Figure 2(d)).

**Figure 3:**
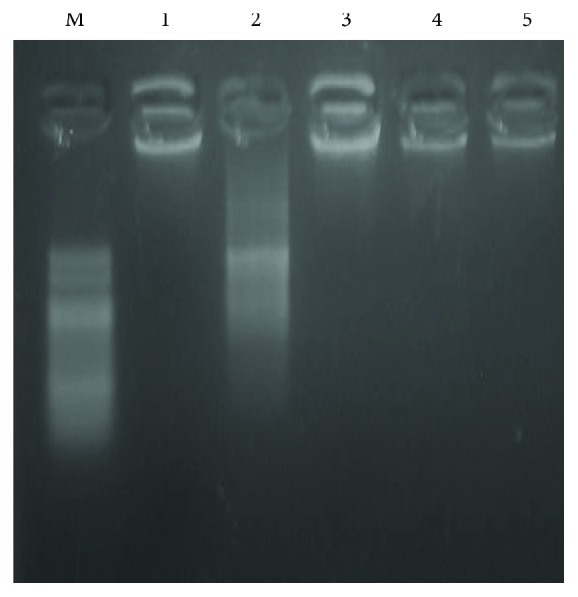
An agarose gel electrophoresis. Image shows DNA fragmentation. Lane M is a DNA marker with 100 bp. Lane 1 shows intact DNA in the saline-treated group. Lane 2 shows DNA streaks of DNA fragmentation in the doxorubicin group. Lanes 3, 4, and 5 show intact DNA in mice treated with BAs (125, 250, or 500 mg/kg) along with DOX. DOX: doxorubicin; BAs: boswellic acids.

Agarose gel electrophoresis for DNA demonstrated normal intact DNA in saline control (group 1) and apoptotic DNA fragments in DOX control (group 2). Groups of mice that received a combination of BAs with DOX (groups 3, 4, and 5) showed less apoptosis and DNA fragmentation when compared to DOX control (group 2) (Figure 3).

**Figure 4:**
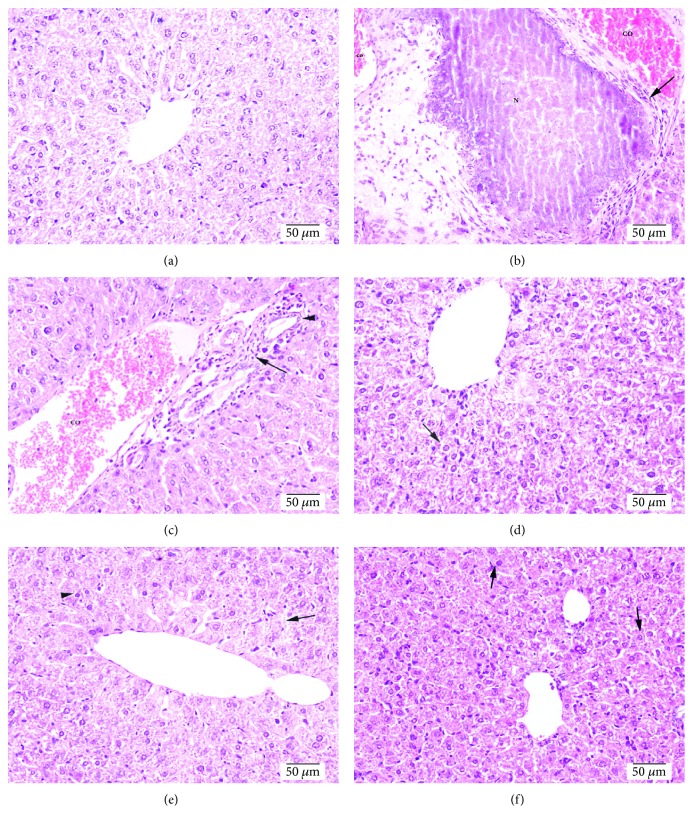
Photomicrographs for hepatic sections stained with hematoxylin and eosin. (a) Section of the liver from the saline group showing normal polyhedral hepatic cells which are arranged in cords that are radically arranged around the central veins. (b) Liver section from the DOX- (6 mg/kg/week) treated group showing marked necrosis of hepatocytes (N), hepatic cell which indicated obscure and fiberized boundary with inflammatory cell infiltration (black arrow), dilatation, and congestion of central veins and portal blood vessels (CO). (c) Section of the liver from the DOX control group showing nuclear pyknosis (white arrow), diffuse vacuolar degeneration (black arrow), and severe congestion of portal blood vessels (co) along with lymphocytic infiltration (black arrow), proliferation of bile duct, and fibrosis. (d) Liver section from mice treated with DOX (6 mg/kg/week) + BAs (125 mg/kg/day) demonstrating hepatic cells showing focal areas of vacuolar degeneration (black arrow). (e) Liver section from mice treated with DOX (6 mg/kg/week) + BAs (250 mg/kg/day) showing normal hepatocytes, mild to moderate vacuolar degeneration (arrow head), and mild hyperemia in the sinusoids (black arrow). (f) Sections from mice treated with DOX (6 mg/kg/week) + BAs (500 mg/kg/day) showing fairly normal tissue architecture and cellular details with mild vacuolar degeneration (black arrow).

Representative hepatic histology for all treatment groups is presented in Figure 4. Normal hepatic sections from saline-treated mice (group 1) showed normal polyhedral hepatic cells which are arranged in cords that are radically arranged around the central veins (Figure 4(a)). However, section from group 2 (doxorubicin control/6 mg/kg/week) showed marked necrosis of hepatocytes (N), hepatic cells with obscure and fiberized boundary with inflammatory cell infiltration (black arrow), dilatation, and congestion of central veins and portal blood vessels (CO) (Figure 4(b)). Moreover, nuclear pyknosis (white arrow), diffuse vacuolar degeneration (black arrow), and severe congestion of portal blood vessels (co) along with lymphocytic infiltration (black arrow), proliferation of bile duct, and fibrosis were very clear (Figure 4(c)). Sections from the liver of DOX + BAs- (125 mg/kg/day) treated group (group 3) showed hepatic cells with focal areas of vacuolar degeneration (black arrow) (Figure 4(d)).

Sections from the liver of group 4 (DOX + BAs/250 mg/kg/day) which revealed normal hepatocytes and vacuolar degeneration ranging from mild to moderate degree (arrow head) and mild hyperemia in the sinusoids (black arrow) are shown in Figure 4(e). On the other hand, Figure 4(f) shows sections from the liver of group 5 (DOX + BAs/500 mg/kg/day) revealing normal tissue architecture and cellular details with few mild vacuolar degeneration (black arrow).

Scoring of histopathological finding of H and E-stained liver section in group 2 (DOX control) demonstrated the greatest score for necrosis, lymphocytic infiltration, nuclear pyknosis, vacuolar degeneration, and congested blood vessels with a total score equal to 18.67 comparing to 0.00 in group 1 (saline control). In groups 3 and 4 (DOX + BAs/125 or 250 mg/kg/day), moderate degrees of necrosis, lymphocytic infiltration, nuclear pyknosis, vacuolar degeneration, and congested blood vessels were observed with a total score equal to 8.67 and 4.67, respectively. Meanwhile, group 5 which is treated with the highest dose of BAs (500 mg/kg/day) in combination with DOX expressed a mild level of necrosis, lymphocytic infiltration, nuclear pyknosis, vacuolar degeneration, and congested blood vessels with a total score equal to 0.67 (Table 2).

## 4. Discussion

A few specialists propose that 66% of natural plants especially therapeutic ones have an incredible antioxidant potential [25]. Acetylation of cellular proteome linking to proapoptotic domain could be changed by ROS in different experiments [26]. BAs are known as mitigating agents against atherosclerosis, hepatotoxicity, and hyperlipidemia [27] and known for their antioxidant and anti-inflammatory activities [1]. Therefore, BAs are considered promising agents in protection against toxic insults involving generation of ROS. Doxorubicin is a standout among the most broadly utilized anticancer drugs, showing action against a wide assortment of tumors. Nonetheless, its symptoms and critical toxicities display a major problem in cancer treatment that requires careful administration and close monitoring for patients' health. This incited us to address in more prominent subtle elements the part and defensive system of BAs in DOX-initiated liver oxidative damage and the resulting improvement of hepatic injury in mice. Doxorubicin-induced hepatotoxicity is well documented in a variety of animal models [5, 28–30]. And, although it might be comparatively minor from a clinician's perspective than its well-established cardiotoxicity, indeed, it still represents a major problem knowing that DOX is extensively metabolized by the liver to the major metabolite doxorubicinol and several hepatotoxic aglycone metabolites [31, 32]. In our study, we selected a dosage regimen and an administration schedule that was previously used by Ali et al. [20] and was proven to cause cardiac toxic effects alongside the antitumor effects.

In the present work, the DOX-treated group prompted increments in the activity of ALT and AST enzymes as compared to the saline-treated group representing clinically significant liver damage that was further confirmed by our histological results. This finding was consistent with several past reports on DOX-prompted hepatotoxicity and apoptosis in patients enduring some forms of liver injury upon DOX administration [33, 34]. The exact mechanism of DOX-prompted hepatotoxicity is not totally explained. Most reviews support the free radical-induced oxidative stress mechanism, as it can be interpreted by the chemical structure of DOX possessing a tendency to generate superoxide anions and peroxynitrite radicals during hepatic drug metabolism [35]. This evokes ROS-initiated lipid peroxidation favoring hepatocyte damage and creating ALT and AST spillage into serum.

This work has investigated specific exposure responses, including focal infiltration by inflammatory cells, proliferation of bile duct, and fibrosis on liver biopsies demonstrating that DOX induces liver harm. These histopathological changes together with the elevated liver enzyme activities recorded in the DOX-treated group are significantly decreased in the DOX- and BAs-treated groups, suggesting protection from the cell damage produced by DOX. Speculations in regard to the mechanism underlying DOX hepatic damage may likewise lay on lessened hepatic HO-1, Nrf2 protein expression, and elevated MDA level favoring oxidative stress with hepatocyte apoptosis.

Here, these mechanisms were initially proposed to be part of the downregulation of hepatic Nrf2 expression. Firstly, reduced glutathione is able to react with cysteine residues in proteins to form disulfides and this chemical process is known as S-glutathionylation [36]. Curiously, S-glutathionylation can modulate Nrf2 gene expression [37]. Under stress conditions, hepatic metabolites upregulate Nrf-2 expression. Further, hepatic gene expression of many antioxidant and phase II/conjugation enzymes involving HO-1 is primarily induced by Nrf-2 tied to ARE/EpRE after being released from Keap1 and translocated to the nucleus [38–40]. And as presented in our results, Nrf2/HO-1 protein has been highly expressed in group 1 (saline group) which can be attributed to the fact that even in normal cells, ROS are produced but in a controlled fashion to help in different physiological processes within the cell [41]. However, the molecular mechanism of action of Nrf2 in the regulation of physiological oxidative stress, detoxification, and removal of numerous exogenous and some endogenous chemicals is expected to be very well functioning in normal cells [42]. Additionally, the expression of Nrf2 has been observed throughout the human tissue, with high expression in detoxification organs, especially the liver [43]. Steady with many lines of confirmation [38, 44] that underscored, antioxidant enzymes were actuated in response to mild oxidative stress. Yet insignificant cellular damage could be known as chemotherapy for cancer treatment. Hereinafter, it was referred to that HO-1 has been downregulated in response to potent ROS production by DOX as noted in many different experimental models [45–47]. Overall, our data highlighted that elevated MDA and downregulated Nrf2/HO-1 protein expression in DOX-treated mice was ameliorated by BAs coadministration. An effect was significantly demonstrated with the three doses of BAs. This finding might be ascribed to free radical-scavenging properties of BAs that are in concurrence with those got by others [48].

Bearing in mind these data, another proposed mechanism of DOX-induced hepatotoxicity may be attributed to hepatocytes' apoptosis that is confirmed in this study by DNA gel electrophoresis showing significant DNA degradation in the doxorubicin-challenged group compared to normal in addition to increased caspase-3 protein expression. A toxic effect was significantly attenuated by the addition of BAs in all used doses (125, 250, and 500 mg/kg/day). The observed DNA damage is in concurrence with that reported previously [49]. Indeed, the very well-known lipophilic properties of DOX and its DNA-binding capacity are responsible for the high concentrations accumulated in hepatic nucleus favoring DNA damage [34]. Unlike the past outcomes highlighting that DOX treatment did not invigorate hepatic caspase-3, caspase-8, and caspase-9 activation or PARP-1 cleavage [50], our data and others suggest that DOX actuates apoptosis signaling in response to ROS production [20, 51].

Taking altogether, we thus find that BAs are valuable in minimizing DOX-promoted hepatic oxidative damage. BAs downregulated hepatic MDA level and upregulated Nrf-2 protein expression and afterward initiated HO-1 expression. Additionally, it provokes geno-defensive impact by downregulating cleaved caspase-3 protein expression which in turn limits DNA fragmentation. Therefore, this complementary medicinal plant can adequately restrain hepatic damage in DOX-treated mice. It might be noted that some of the investigated parameters were ameliorated in a dose-response manner (e.g., Nrf-2, HO-1, and cleaved caspase-3) while others were not (e.g., MDA) which might be attributed to being more sensitive to the antioxidant effect of BAs than the others. Supporting our work, data published by Kruger et al. [52] have detailed that the metabolic active elements of BAs go about as antioxidants and chelate metals involved in oxidative stress pathways. Another clarification is managed by Hartmann et al. [9] who proposed that BAs possess antioxidant effects restraining MDA production in acute experimental colitis. In addition, previous findings accentuated that BAs evoke antioxidant properties that fortify the Nrf2/HO-1 defense pathway [8, 14].

Since the past works highlighted the stimulant action of BAs on the Nrf2/HO-1 pathway in addition to the inhibitory impacts on MDA production and caspase-3 expression, along these lines, we here can depict the hepatic apoptotic inhibitory impact of BAs by attenuating both initiation and execution phases of apoptosis through its antioxidant properties.

Finally, perhaps one of the considerations when coadministering DOX and an antioxidant agent is that whether this agent would interfere with the desired tumor cell death. However, it has been documented that DOX toxicity in cancer cells primarily occurs through DNA intercalation and damage [53], whereas its cardiotoxicity or hepatotoxicity mainly occurs by generating oxygen free radicals, which can be inhibited by free radical scavengers [54–56]. This difference in DOX-mediated toxicity in cancer and normal cells can be used to improve the antitumor effects of DOX with combinatorial approaches that allow protecting normal cells without affecting the desired oncolytic activity. Another investigated concept is that the defensive mechanisms of many cancer cells are known to be impaired. This makes tumor cells unable to utilize the extra antioxidants in a repair capacity leading to cell death [57]. Furthermore, if we consider the induction of Nrf2 expression in cancer cells by BAs, this will increase the chemosensitivity of anticancer agents since Nrf2 is widely debated for a dual role in chemosensitive and chemoprotective mechanisms [58].

## 5. Conclusions

Oxidative stress is viewed as the real occasion fundamental DOX hepatic harmfulness. Our outcomes in this study propose that doxorubicin administration is joined by indications of oxidative damage, including elevated hepatic MDA level in accordance with DNA fragmentation, caspase-3 protein expression, HO-1, and Nrf2 protein downregulation. In this work, BAs were capable of deactivating ROS, repressing lipid peroxidation and DNA damage. We also highlighted that the antioxidant effects of BAs involve the regulation of the HO-1/Nrf2 defense pathway and subsequently protect the liver from DOX-induced toxicity. Further studies are also needed to test the corresponding terminal doxorubicin blood concentrations across the dose groups to demonstrate consistency of exposure to strengthen follow-up studies.

## Figures and Tables

**Table 1:** Effect of three weekly doses of doxorubicin (6 mg/kg) alone or in combination with boswellic acids (125, 250, or 500 mg/kg/day) on serum ALT and AST in mice.

Groups	ALT (unit/l)	AST (unit/l)
Saline	59.17 ± 14.39 (%CV = 24.31)	84.17 ± 17.41 (%CV = 20.68)
Doxorubicin (6 mg/kg/week)	158.83 ± 28.14^a^ (%CV = 17.72)	191.33 ± 45.26^a^ (%CV = 23.66)
Doxorubicin (6 mg/kg/week) + BAs (125 mg/kg)	110.83 ± 22.46^b^ (%CV = 20.27)	158.17 ± 27.59^b^ (%CV = 17.44)
Doxorubicin (6 mg/kg/week) + BAs (250 mg/kg)	91.33 ± 8.71^b^ (%CV = 9.54)	129.33 ± 18.03^b^ (%CV = 13.94)
Doxorubicin (6 mg/kg/week) + BAs (500 mg/kg)	72.2 ± 12.81^bc^ (%CV = 17.79)	125.67 ± 10.78^bc^ (%CV = 8.58)

Mice were injected with doxorubicin (DOX, 18 mg/kg i.p.) in combination with boswellic acids (BAs, 125, 250, or 500 mg/kg). Data are mean ± SD, and analysis was done by one-way ANOVA followed by Tukey's post hoc test. ^a^Compared to the saline group. ^b^Compared to the DOX group. ^c^Compared to the doxorubicin + BAs (125 mg/kg) group. *P* value < 0.05.

**Table 2:** Histopathological score of hepatic tissues from mice treated with three weekly doses of doxorubicin (6 mg/kg) alone or in combination with boswellic acids (125, 250, or 500 mg/kg/day). Scoring for hepatic tissues stained with hematoxylin and eosin was performed as 0 = absent; 1 = low or weak; 2 = mild; 3 = moderate; and 4 = high or frequent, and the total score was calculated from these. Presented results are the mean ± SD and were analyzed using one-way ANOVA followed by Bonferroni's post hoc test at *P* < 0.05.

Groups	Necrosis	Lymphocytic infiltration	Nuclear pyknosis	Vacuolar degeneration	Congested blood vessels	Total score	Mean ± SD
Saline	0.00 ± 0.00	0.00 ± 0.00	0.00 ± 0.00	0.00 ± 0.00	0.00 ± 0.00	**0**	0.00 ± 0.00
Doxorubicin (6 mg/kg/week)	3.67 ± 0.58^a^ (%CV = 15.8)	3.33 ± 1.15^a^ (%CV = 34.5)	3.67 ± 0.58^a^ (%CV = 15.8)	4.00 ± 0.00^a^ (%CV = 0.00)	4.00 ± 0.00^a^ (%CV = 0.00)	**18.67** ^**a**^	3.73 ± 0.59^a^ (%CV = 15.8)
Doxorubicin (6 mg/kg/week) + BAs (125 mg/kg)	1.67 ± 0.58^b^ (%CV = 34.7)	1.33 ± 0.58^b^ (%CV = 43.6)	2.00 ± 1.00^b^ (%CV = 50)	2.00 ± 0.00^b^ (%CV = 0.00)	1.67 ± 0.58^b^ (%CV = 34.7)	**8.67** ^**b**^	1.73 ± 0.59^b^ (%CV = 34.1)
Doxorubicin (6 mg/kg/week) + BAs (250 mg/kg)	0.67 ± 0.58^c^ (%CV = 86.6)	1 ± 0.00^b^ (%CV = 0.00)	0.67 ± 0.58^c^ (%CV = 86.6)	1.33 ± 0.58^c^ (%CV = 43.6)	1 ± 1.00^b^ (%CV = 100)	**4.67** ^**c**^	0.93 ± 0.59^c^ (%CV = 34.1)
Doxorubicin (6 mg/kg/week) + BAs (500 mg/kg)	0.00 ± 0.00 (%CV = 0.00)	0.00 ± 0.00 (%CV = 0.00)	0.00 ± 0.00 (%CV = 0.00)	0.67 ± 0.58^d^ (%CV = 86.6)	0.00 ± 0.00 (%CV = 0.00)	**0.67** ^**d**^	0.13 ± 0.35^d^ (%CV = 269.2)

^a^Compared to the saline group. ^b^Compared to the DOX group. ^c^Compared to the doxorubicin + BAs (125 mg/kg) group. ^d^Compared to the doxorubicin + BAs (250 mg/kg) group. *P* value < 0.05.

## Abbreviations

ALT: Alanine aminotransferase
AST: Aspartate aminotransferase (AST)
BAs: Boswellic acids
DOX: Doxorubicin
MDA: Malondialdehyde
Nrf2: Transcription factor/heme oxygenase-1 (Nrf2/HO-1)
ROS: Reactive oxygen species.
